# Queen's Jubilee Hospital

**Published:** 1905-07-15

**Authors:** 


### QUEEN'S JUBILEE HOSPITAL.

It will be remembered that this hospital, on March 13 last, requested King Edward's Hospital Fund to inquire into the recent trouble which resulted in the resignation of the late medical staff and the appointment of a new one. The report and recommendations of the King's Fund, dated April 10th have already been published, and also the reply of the board of management, dated May 10th, in which they expressed their disagreement with the findings of the King's Fund, and made alternative recommendations. The following correspondence has since taken place :—

81 Cheapside, E.C., June 3rd, 1905.

Miss H. S. Lane, Secretary, Queen's Jubilee Hospital,
Richmond Road, Earl's Court, S.W.

Madam,—Referring to your letter of May 15th enclosing a printed statement relating to the report of the inquiry held at the request of your board by this fund into the affairs of the Queen's Jubilee Hospital, I am desired by the executive committee to say that it appears to them the matter now rests with the governors of the hospital. Our recommendations were in no sense demands, but were merely offered as advice. If the governors prefer taking some other course we shall not in any way resent their doing so, and we earnestly hope that whatever is decided on will accomplish the desired object—namely, the good of the hospital—which we recognise to the full has been the only object of the members of the board of management in freely giving time, which many of them could ill afford to spare, for a public object. I am, however, desired to add for your information that this fund would not be disposed to nominate any members of a board which was partly elected by another body. The main object of our recommendations, to which we still adhere, was to place a period of, if possible, 12 months between the present and future management of the hospital. In order to provide for this we should have appointed independent gentlemen residing in the neighbourhood, but unconnected with the hospital or this fund, and we should only feel justified in making an extraordinary grant for the expenses under those special circumstances.

Yours faithfully,
John Craggs, Honorary Secretary.

The Queen's Jubilee Hospital, Earl's Court,
London, S.W. July 5, 1905.

J. Danvers Power, Esq.

Dear Sir,—I am directed by the board of management to inform you that a meeting of the governors of the Queen's Jubilee Hospital was held on June 29 to consider the report of the executive of King Edward's Hospital Fund, and the board's reply thereto. I am to state that the feeling of the meeting was that, having given full consideration to the recommendations of the King's Fund, the governors felt that the board of management had adopted the best course open to them, and it was resolved that the board's reply to the King's Fund be formally approved and adopted. I am further instructed to say that the board of management express the hope that the executive of the King's Fund will continue to accord the hospital their valuable support.

I am, dear Sir, yours faithfully,
H. S. Lane, Secretary.

81 Cheapside, E.C. July 7, 1905.

Miss H. S. Lane, Secretary, Queen's Jubilee Hospital,
Richmond Road, Earl's Court, S.W.

Dear Madam,—I beg to acknowledge receipt of your letter of the 5th inst. As already stated, our recommendations were offered as advice, to which we still adhere. Applications for grants from the Queen's Jubilee Hospital will, as heretofore, be considered on their merits, as in the case of every other hospital.

Yours faithfully,
J. Danvers Power, Honorary Secretary.

The sum offered by the committee of the King's Fund, in the event of their advice being accepted, was £1,000.

